# Dysregulated Plasma Membrane Turnover Underlying Dendritic Pathology in Neurodegenerative Diseases

**DOI:** 10.3389/fncel.2020.556461

**Published:** 2020-10-06

**Authors:** Chang Geon Chung, Sung Soon Park, Jeong Hyang Park, Sung Bae Lee

**Affiliations:** Department of Brain and Cognitive Sciences, Daegu Gyeongbuk Institute of Science and Technology (DGIST), Daegu, South Korea

**Keywords:** plasma membrane turnover, dendritic pathology, neurodegenerative diseases, Rab GTPases, dendritic secretory pathway, dendritic endocytic pathway

## Abstract

Due to their enormous surface area compared to other cell types, neurons face unique challenges in properly handling supply and retrieval of the plasma membrane (PM)—a process termed PM turnover—in their distal areas. Because of the length and extensiveness of dendritic branches in neurons, the transport of materials needed for PM turnover from soma to distal dendrites will be inefficient and quite burdensome for somatic organelles. To meet local demands, PM turnover in dendrites most likely requires local cellular machinery, such as dendritic endocytic and secretory systems, dysregulation of which may result in dendritic pathology observed in various neurodegenerative diseases (NDs). Supporting this notion, a growing body of literature provides evidence to suggest the pathogenic contribution of dysregulated PM turnover to dendritic pathology in certain NDs. In this article, we present our perspective view that impaired dendritic endocytic and secretory systems may contribute to dendritic pathology by encumbering PM turnover in NDs.

## Introduction

Dendrites are neuronal compartments essential for receiving electrochemical signals from presynaptic neurons through formed synapses. Accurate neuronal wiring relies critically on the proper establishment of the dendritic field that is achieved by both structural build-ups of dendritic arbors and functional maturation of synapses (Jan and Jan, 2010). The establishment of the dendritic field is by nature a dynamic process as it is inevitably accompanied by dramatic changes in the morphology of entire dendritic arbors. Even after the establishment of the dendritic field, neuronal connections can be rewired in response to changes in the external environment by dynamically altering dendritic morphology and readjusting formed synapses. Therefore, disruption of dendritic morphology will invariably lead to failed synapse formation and communication between neurons.

To maintain dendritic morphology and dynamics, neurons need a constant turnover of plasma membranes (PMs). This process of PM turnover is mediated primarily by endocytic and secretory pathways. However, due to its highly elaborate dendrites, a typical neuron has a 10,000 times larger surface area than does a typical epithelial cell (Horton and Ehlers, 2004). Thus, a neuron will undoubtedly face a staggering challenge to grow and maintain those dendrites if it were to rely solely on somatic endocytic and secretory systems (Pfenninger, 2009). Thankfully, neuronal dendrites showcase various types of endocytic and secretory components, which participate in dendritic growth and maintenance, as well as a local supply of PM proteins (Jan and Jan, 2010; Puram and Bonni, 2013; Kennedy and Hanus, 2019).

Dendritic changes are frequently observed in animal models of various neurodegenerative diseases (NDs), such as Alzheimer’s disease (AD), Parkinson’s disease (PD), polyglutamine (polyQ) diseases, and amyotrophic lateral sclerosis (ALS; summarized in Table 1). Consistently, dendritic pathology has been reported in post-mortem brain samples of patients with these diseases (Mehraein et al., 1975; Graveland et al., 1985; Nakano and Hirano, 1987; Patt et al., 1991; Ferrer, 1999; Kulkarni and Firestein, 2012). Although affected neuronal cell types and the patterns of dendritic changes vary depending on the disease, NDs generally share common pathological features such as decreased dendritic complexity and impaired synaptic maturation (Kulkarni and Firestein, 2012; Herms and Dorostkar, 2016). Previous studies identified several molecules and cellular processes involved in dendritic pathology in NDs. For example, a recent study identified a transcription factor Forkhead Box O (FOXO) whose sequestration by nucleus-accumulated toxic polyQ proteins in *Drosophila* sensory neurons results in dendritic defects (Kwon et al., 2018). In AD, β-amyloid (Aβ) has been reported to cause dendritic spine loss and to decrease expression of AMPA receptor on the synaptic surface by enhancing endocytosis in CA1 pyramidal neurons (Hsieh et al., 2006). In a PD model, knockout of *Pink1* showed a shortening of dendritic lengths presumably through disrupting mitochondrial transport in mouse primary cortical and midbrain neurons (Dagda et al., 2014). In a UBQLN2-P497H mouse model of ALS, impairment of the protein degradation system led to dendritic spinopathy accompanied by synaptic dysfunction, and cognitive deficits (Gorrie et al., 2014). Besides what we have described so far, many other molecules have been identified whose dysregulation interferes with cellular components such as cytoskeletons, mitochondria, endosomes, ER, and Golgi that may be linked to dendritic pathology (Jan and Jan, 2010; Lei et al., 2016; Kweon et al., 2017; Kelliher et al., 2019). Currently, how these cellular components contribute to dendritic pathology is being worked out in many labs. Here, we propose that dendritic endocytic and secretory pathways, when disrupted, may contribute to dendritic pathology in several NDs.

**Table 1:** Dendritic pathology characterized in animal models of neurodegenerative diseases (NDs).

Neurodegenerative diseases (NDs)	Disease models tested	Phenotypes	Species	Neuronal cell types	References
Alzheimer’s disease (AD)	APP-695 O/E	Decreased dendritic spine density.	Mouse	CA1 pyramidal neurons	Hsieh et al. (2006)
	APP-K670N/ M671L, PS1 M146V O/E	Decreased dendritic length, dendritic surface area, and numbers of dendritic branches.	Mouse	CA1 pyramidal neurons	Šišková et al. (2014)
	APP-695 O/E	Decreased dendritic spine density and increased dendritic spine elimination.	Mouse	Cortical neurons	Spires et al. (2005); Spires-Jones et al. (2007)
	APP-OSK O/E	Loss of dendritic spines.	Mouse	Hippocampal neurons	Umeda et al. (2015)
	Tau-P301L O/E	Degeneration of dendrites.	Mouse	CA1 pyramidal neurons	Jaworski et al. (2011)
	Tau-P301S O/E	Decreased dendritic spine density.	Mouse	Cortical pyramidal neurons	Hoffmann et al. (2013)
Parkinson’s disease (PD)	LRRK2-G2019S O/E	Dendritic degeneration.	Fly	Dendritic arborization neurons	Lin et al. (2010)
	PINK1 KO	Decreased dendritic length.	Mouse	Primary cortical neurons	Dagda et al. (2014)
	SNCA-A30P O/E	Decreased branching of dendritic spines.	Mouse	Adult-born granule cells	Neuner et al. (2014)
Huntington’s disease (HD)	Htt-47Q O/E	Loss of dendritic spines.	Mouse	Primary hippocampal neurons	Richards et al. (2011)
	Htt-69Q O/E	Decreased number of dendritic spines.	Mouse	Cortical/hippocampal neurons	Murmu et al. (2013)
	Htt-115Q O/E	Decreased number of dendritic spines.	Mouse	Medium spiny neurons and pyramidal neurons	Spires et al. (2004)
Amyotrophic lateral sclerosis (ALS)	SOD1-G93A O/E	Increased dendritic arbor length in early stages and decreased dendritic arbor length in late stages.	Mouse	Brainstem XII MNs	Fogarty et al. (2017)
		Decreased dendritic length.	Mouse	Lumbar spinal cord MNs
	TDP-43 KD	Decreased dendritic branches and complexity.	Mouse	Primary hippocampal neurons	Schwenk et al. (2016)
	C9orf72 KO	Decreased dendritic arborization and spine density.	Mouse	Primary hippocampal neurons	Ho et al. (2019)
	UBQLN2-P497H O/E	Decreased density of dendritic spines.	Mouse	Granule cell layer of the dentate gyrus	Gorrie et al. (2014)
Frontotemporal dementia (FTD)	CHMP2B-Intron5 O/E	Increased dendritic branches.	Mouse	Primary cortical neurons	Clayton et al. (2018)

In this review article, we will first describe the general mechanisms of PM turnover mediated by endocytosis and exocytosis. Next, we will provide an overview of dendritic endocytic and secretory pathways. Afterward, we will discuss how local molecular machinery might regulate dendritic endocytic and secretory pathways for PM turnover and how their dysfunction might contribute to dendritic pathology in several NDs. Finally, we will propose how dendritic endocytic and secretory pathways might be linked to selective dendritic vulnerability in NDs.

## Basic Mechanisms of PM Turnover in Neurons: Endocytosis and Exocytosis

PM turnover is defined as the process by which membranes are continuously cycled to and from the PM. Through this process a cell can: (1) expand or reduce its size; (2) alter its shape; and (3) insert or remove from its PM the membranous lipids and proteins needed to convey both intra- and extra-cellular signals.

How is PM turnover regulated in neurons? Exocytosis and endocytosis are thought to be the primary means by which expansion and retrieval of the PM are mediated, respectively (Pfenninger, 2009; Peng et al., 2015). In a typical cell, materials that comprise the PM are first synthesized in the endoplasmic reticulum (ER) and then are modified and sorted in Golgi, from where vesicles bud and are inserted into the PM by exocytosis. In yeast, exocytosis of these PM-expanding vesicles requires tethering to PM by exocyst, without which soluble *N*-ethylmaleimide-sensitive factor attachment protein receptor (SNARE) complexes required for the membrane fusion do not form (TerBush et al., 1996; Grote et al., 2000). In neurons, their contribution to the growth of neurites (Vega and Hsu, 2001; Murthy et al., 2003)—and more specifically dendrites (Peng et al., 2015; Zou et al., 2015; Lira et al., 2019)—has been observed in *Drosophila* and cultured mammalian neurons. Interestingly, exocyst seems to be dispensable for neurotransmitter secretion in *Drosophila* (Murthy et al., 2003; Mehta et al., 2005), but not in primary hippocampal neurons (Lira et al., 2019). Generally, for membranes to fuse, SNARE proteins must be present on both membranous systems (Südhof and Rothman, 2009). For instance, Urbina et al. (2018) showed that VAMP2-positive exocytic vesicles contribute to PM expansion in neurites of mouse cortical neurons. Another SNARE protein, tetanus neurotoxin-insensitive (TI)-VAMP, has been shown to contribute to both axonal and dendritic growth without affecting synaptic vesicle fusion in primary neuronal cultures (Coco et al., 1999; Martinez-Arca et al., 2000, 2001). However, a knockout of *TI-VAMP* in mice only partially limited neurite outgrowth, suggesting that other SNARE proteins may mediate PM expansion (Meldolesi, 2011; Sato et al., 2011). In 2014, another group showed that the exocytosis of VAMP4-positive vesicles seems to contribute to the neurite growth in PC12 cells (Colombo et al., 2014). Interestingly, another SNARE protein, Sec22b, has been shown to contribute to PM expansion in neurons probably by mediating lipid transfer from ER to PM without vesicular fusion (Petkovic et al., 2014).

**Figure 1:**
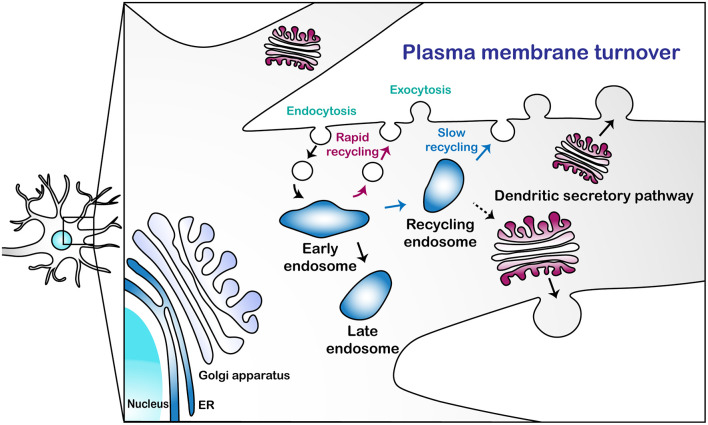
A schematic illustration of the plasma membrane turnover in neuronal dendrites. This illustration describes three pathways for plasma membrane (PM) turnover: rapid endosomal recycling *via* early endosomes (EEs); slow endosomal recycling *via* recycling endosomes (REs); and secretion *via* the dendritic secretory pathway.

Endocytosis is the primary means by which PM is internalized, which may offset the functions of exocytosis. In *Drosophila* C4 dendritic arborization (da) neurons, defects in exocytosis-mediated dendritic growth were mitigated by blocking clathrin-mediated endocytosis (CME) using a temperature-sensitive dominant-negative allele of *shibire* (*shi^ts1^*, Peng et al., 2015). Urbina et al. (2018) also showed that CME contributes to the retrieval of PM in shaping neurite growth in mouse cortical neurons and suggested that exocytosis-mediated PM expansion in neurites can be counterbalanced by CME. Some of these endocytic vesicles, once internalized *via* endocytosis, may directly fuse with *medial*/*trans*-Golgi, at least in yeast (Day et al., 2018). In general, however, most other endocytic vesicles fuse with early endosomes (EEs), wherein sorting of the PM components takes place. Those components may be rapidly recycled back to the PM from EEs or slowly *via* recycling endosomes (REs; Taguchi, 2013). As EEs mature into late endosomes (LEs) *en route* to degradation (Huotari and Helenius, 2011), some of the PM components are recycled back to Golgi *via* retromers (Chen et al., 2019). Collectively, these two processes—endocytosis and exocytosis—regulate PM turnover in a typical cell, likely including neurons (Figure 1).

## The Presence of Dendritic Endocytic and Secretory Pathways and Their Potential Link to Dendritic Morphology in Neurons

Although it is fairly well established that the endocytic and secretory pathways, in general, regulate dendritic morphology *via* PM turnover, the extent to which dendritic endocytic and secretory pathways partake in regulating dendritic morphology *via* local PM turnover is less clear. Although the endocytic organelles have been fairly well delineated in dendrites, secretory organelles in dendrites have remained more elusive. Here, we will briefly discuss several dendritic endocytic and secretory units and their potential relevance to dendritic morphology.

### Dendritic Endocytic Pathway

All the major types of endosomes—EEs, LEs, and REs—have been shown to exist in dendrites. Endocytosis in dendrites has been reported to play a major role in *Drosophila* dendritic pruning by triggering dendritic thinning *via* internalizing PM (Kanamori et al., 2015) and through internalizing specific cell adhesion molecule, Neuroglian (Zhang et al., 2014). Although endocytosis is one of the means by which EEs are produced (Mellman, 1996), blocking endosomal transport from soma to dendrites leads to depletion of EEs in dendrites of *Drosophila* da neurons (Satoh et al., 2008; Zheng et al., 2008), suggesting that most of the EEs in dendrites may be derived from the soma. Intriguingly, a recent study shows that the trans-Golgi network (TGN), but not endocytosis, is indispensable in forming Rab5-labeled EEs in yeast (Nagano et al., 2019). However, whether EEs can be synthesized from TGN in neurons, let alone neuronal dendrites, has not been shown.

LEs and REs, which are thought to be derived from EEs (Mellman, 1996), are also detected in dendrites and/or dendritic spines (Cooney et al., 2002; Cheng et al., 2018; Yap et al., 2018). LEs are well known for their role in sorting ubiquitinated proteins for degradation *via* lysosomes (Hu et al., 2015). In dendrites, they transport dendritic cargos towards the soma for degradation *via* lysosomes (Cheng et al., 2018; Yap et al., 2018). However, one study showed that LEs translocate to and fuse with the PM after making repeated contact with the ER. This process was shown to contribute to neurite growth in PC12 cells (Raiborg et al., 2015). REs have been shown to mediate PM turnover process in dendrites and dendritic spines, thereby mediating their growth in rat hippocampal or cortical neurons (Park et al., 2006; Bowen et al., 2017). Interestingly, REs have been shown to exchange cargoes and make physical contact with Golgi in *Drosophila*, sea urchin embryos, and mammals (Mallard et al., 1998; Fujii et al., 2020a,b). However, their interaction in neuronal dendrites has not been reported. Further examining the potential interplay between these different types of endosomes and secretory organelles, such as ER and Golgi, in dendrites may provide significant insight on the mechanisms underlying PM turnover.

### Dendritic Secretory Pathway

The ER that localizes in the dendritic spine, called spine apparatus (SA), has a specialized membrane-stacked morphology similar to that of Golgi (Gray, 1959a,b). Based on its morphology, SA was speculated to play a role in dendritic secretory function, though it did not garner any significant experimental support for a long time. In 2001, through post-embedding immunogold labeling in adult rat tissue, Pierce et al. (2001) showed in neuronal dendrites the presence of a repertoire of proteins that localize to ER, ER-Golgi intermediate compartment (ERGIC), and Golgi, indicative of a presence of dendritic secretory pathway. Interestingly, although some of those proteins were found nearby SA in dendritic spines, others were found within dendrites away from SA. In contrast, a couple of subsequent studies were not able to verify this finding in cultured neurons (Hanus et al., 2014; Bowen et al., 2017). Whether or not the difference in sample type is accountable for this apparent discrepancy remains to be tested.

Another distinct secretory organelle found in dendrites is termed Golgi outpost (GOP), which was first defined in cultured hippocampal neurons (Horton and Ehlers, 2003). A correlation between the localization of GOPs at branch points and dendritic growth in both *Drosophila* (Ye et al., 2007; Lin et al., 2015) and mammals (Horton et al., 2005; Ye et al., 2007) supports the purported function of GOPs in dendritic growth *via* PM supply. In the branch points, GOPs have been shown to function in supplying PM proteins, such as BDNF (Horton and Ehlers, 2003), ADAM10 (Saraceno et al., 2014), Kainate receptors (Evans et al., 2017), and NMDA receptors (Jeyifous et al., 2009) in mammalian hippocampal neurons. The potential role of GOPs in the dendritic pathology of neurological disorders has been recently discussed in a review article (Caracci et al., 2019). However, GOP seems to be relatively rare in mammalian neurons (Horton et al., 2005; Hanus et al., 2014; Bowen et al., 2017), and even absent in mouse Purkinje cells 2 weeks after birth (Liu et al., 2017). Therefore, the importance of GOPs in dendritic morphology in adult neurons remains to be further elucidated.

Relatively recently characterized dendritic secretory units further complicate our understanding of the dendritic secretory pathway. ERGIC is normally sandwiched in between ER and *cis*-Golgi but is scattered all over the dendrites of rat hippocampal (Hanus et al., 2014) or cortical neurons (Bowen et al., 2017). In dendrites, ERGIC seems to perform a secretory function, bypassing Golgi entirely (Hanus et al., 2014; Bowen et al., 2017). However, by using a highly specific Golgi marker, pGolt, another study provides evidence that a small (200–1,000 nm in diameter) Golgi membrane compartment, termed Golgi satellite (GS), exists in between ERGIC and retromer in dendrites and participates in local PM turnover in rat hippocampal primary neurons (Mikhaylova et al., 2016). The authors further show that GSs are more numerous than GOPs and are positive for some Golgi markers, but not for all. Considering that *cis*-, *medial*-, and *trans*-Golgi compartments often exist separately in *Drosophila* da neuronal dendrites (Zhou et al., 2014), failure to detect Golgi compartments in dendrites of other types of neurons by some other groups may be due to the simplified structure of dendritic Golgi, which may be missing some structural proteins that are often used to label Golgi, such as GM130 (Zhou et al., 2014). Overall, the dendritic secretory pathway is extremely complicated and there is still much left to be discovered.

## The Local Molecular Machinery That May Regulate Dendritic Endocytic and Secretory Pathways for PM Turnover

### Rab GTPases as Potent Local Regulators of the Endocytic Pathway

Ras-related in brain (Rab) GTPase proteins are among the most compelling candidate molecular machinery that may play crucial roles in (dendritic) PM turnover. Rabs, which were first found in rat brains (Salminen and Novick, 1987), are significantly conserved among eukaryotes from yeast to human (Rojas et al., 2012). To date, more than 60 different Rabs have been identified in humans (Kiral et al., 2018). Rabs are the largest group of proteins in the Ras superfamily and function as molecular switches in diverse cellular contexts (Zhen and Stenmark, 2015); they are master regulators of membrane transport between organelles, or between an organelle and PM (Wandinger-Ness and Zerial, 2014). Given these known generalized functions, Rabs will likely play a major role in neuronal dendritic PM turnover.

#### Regulation of Rab Activity by Switching Its Guanine Nucleotide Status

The activity of Rab is determined by its guanine nucleotide status: GTP-bound is active and GDP-bound is inactive. Regulatory proteins, such as Rab guanine nucleotide exchange factors (Rab GEFs), Rab GTPase-activating proteins (Rab GAPs), and Rab-GDP dissociation inhibitors (Rab GDIs) control the guanine nucleotide status of Rab (Welz et al., 2014). Rab GEFs facilitate the release of GDP from Rabs, which allows them to bind GTP (Stenmark, 2009). GTP-bound active Rabs are then targeted to the particular membrane site where they collect effector proteins, such as sorting adaptors, tethering factors, kinases, phosphatases, and motor proteins, through which vesicle trafficking is mediated between membranous compartments. On the other hand, Rab GAPs catalyze the hydrolysis of GTP into GDP. Subsequently, Rab GDI binds to Rab-GDP, extracts it away from the membrane, and stabilizes this inactive form of Rab in the cytosol by preventing it from releasing GDP.

#### Characterized Roles of Rab GTPases in PM Turnover *via* the Endocytic Pathway

Endosomal membrane trafficking can be broadly divided into two different pathways: PM internalization to endosomal compartments and recycling vesicles from endosomes to PM. In this review article, we briefly explain the roles of various Rabs associated with each pathway (summarized in Table 2).

**Table 2:** Rab GTPases in endocytic pathways.

Endosomal pathways	Rab proteins	Molecular functions	Cellular functions in dendrites	Main effectors	Main cargoes	References
Endocytosis	Rab5	PM to EE vesicle trafficking, vesicle budding, vesicle motility, vesicle uncoating, and vesicle tethering.	Dendritic branching, development, thinning, and pruning	VPS34, EEA1, Rabaptin-5, Rabex-5	TfR, EGFR, B2AR, TrkB	Stenmark et al. (1995), Horiuchi et al. (1997), McLauchlan et al. (1998), Rubino et al. (2000), Leonard et al. (2008), Satoh et al. (2008), Semerdjieva et al. (2008), von Zastrow and Williams (2012), Kanamori et al. (2015), Zhang et al. (2014), Wang et al. (2017), and Moya-Alvarado et al. (2018)
Exocytosis (Rapid recycling)	Rab35	EE to PM vesicle trafficking.	Neurite outgrowth	EPI64C, ACAP2, MICAL-L1	TfR, TCR	Kouranti et al. (2006), Patino-Lopez et al. (2008), Kobayashi and Fukuda (2013), and Kobayashi et al. (2014)
	Rab4	EE to PM vesicle trafficking.	Dendritic branching and development; Spine formation	Arl1, BIG1, BIG2, GRASP-1, NBEA	TfR, GPCRs, AMPA receptor, NMDA receptor	Seachrist et al. (2000), Seachrist and Ferguson (2003), Odley et al. (2004), Zheng et al. (2008), Yudowski et al. (2009), Hoogenraad et al. (2010), Esseltine et al. (2011), D’Souza et al. (2014), and Gromova et al. (2018)
Exocytosis (Slow recycling)	Rab11	Vesicle trafficking and motility from EE to RE or from RE to PM; RE biogenesis.	Dendritic branching and pruning; Spine growth	Rab11-FIPs, MyoVb, Dynein, Sec15	AMPA receptor, TfR, EGFR, TLR4, β1 integrin, N-cadherin, E-cadherin, TrkB	Ullrich et al. (1996), Sönnichsen et al. (2000), Park et al. (2004), Zhang et al. (2004), Park et al. (2006), Wang et al. (2008), Horgan et al. (2010), Kelly et al. (2012), Lazo et al. (2013), Kramer et al. (2019), and Lin et al. (2020)
	Rab22	EE to RE vesicle trafficking; RE biogenesis.	N/A	N/A	TfR, CD1a, MHC class1, TrkA	Weigert et al. (2004), Magadan et al. (2006), Barral et al. (2008) and Wang et al. (2011)
	Rab25	EE to RE vesicle trafficking; Vesicle sorting between RE and LE.	N/A	N/A	IgA, TfR, β1 integrin	Casanova et al. (1999), Wang et al. (2000), Caswell et al. (2007), Dozynkiewicz et al. (2012), and Jeong et al. (2019)
	Rab10	EE to RE vesicle trafficking; Vesicle motility; RE biogenesis.	Dendritic development and branch patterning	Kif13A/B	CD147, TfR	Chen et al. (2006), Taylor et al. (2015), Zou et al. (2015), and Etoh and Fukuda (2019)
	Rab8	RE to PM vesicle trafficking.	Dendritic spine growth	N/A	AMPA receptor, TfR	Hattula et al. (2006) and Brown et al. (2007)

#### PM to EE

Retrieval of the PM is mediated by the internalization of a portion of PM, mostly *via* CME (Bitsikas et al., 2014). The endocytic vesicles are then targeted to EEs by Rab5. PM proteins, such as Transferrin Receptor (TfR), epidermal growth factor receptor (EGFR; Leonard et al., 2008), and β-2-adrenergic receptor (β2AR; von Zastrow and Williams, 2012), are reported to be transported to EEs *via* this endocytic pathway.

How Rab5 regulates this endocytic pathway is relatively well known. First, Rab5-GDI and adaptor protein 2 (AP2) complexes initiate vesicle budding from PM at clathrin-coated pits (McLauchlan et al., 1998). Rab5-vesicles then uncoat AP2 adaptor complexes and coat proteins, a process required for vesicle fusion with EEs (Semerdjieva et al., 2008). Lastly, Rab5 recruits various effectors, such as VPS34, EEA1, and Rabaptin-5/Rabex-5 complex (Stenmark et al., 1995; Horiuchi et al., 1997; Rubino et al., 2000), through which the endocytic vesicles dock and fuse with EE membrane.

#### Endosomes to PM

The PM is recycled mostly through two distinct endosomal pathways: the rapid recycling (1–5 min) pathway, through which membrane vesicles are transported directly from EEs to PM; and the slow recycling (10–20 min) pathway, through which membrane vesicles are transported to PM *via* REs (Jonker et al., 2020).

##### Rapid Recycling

TfR is among the well-characterized membrane proteins that go through the rapid recycling pathway. This pathway is known to be selectively blocked by knockdown or knockout of *Rab35* (Kouranti et al., 2006), or by overexpression of its dominant-negative form (Patino-Lopez et al., 2008). In addition to Rab35, Rab4 also plays a crucial role in the regulation of TfR recycling *via* the rapid recycling pathway. Rab4 is primarily located around the exit sites of EE (EEES), where membrane fission actively occurs (Stenmark, 2009). At EEES, Rab4 recruits effectors, thereby promoting the Class I ARF cascade. It has been shown that inhibition of Rab4 effectors disrupts the elongated tubular formation of EE, an important process in the rapid recycling pathway (D’Souza et al., 2014). Consistently, when Rab4 was inhibited by overexpressing its dominant-negative form in HEK293 cells, TfR rapid recycling was perturbed (Yudowski et al., 2009).

##### Slow Recycling

RE is defined as a membranous compartment positive for Rab11 (Grant and Donaldson, 2009). Fluorescent live imaging shows that RE is generated by tubule elongation of EE, from which Rab5 gradually disappears and is replaced by Rab11 (Sönnichsen et al., 2000). Rab11 works together with numerous other Rabs and their effectors to engage in the overall process of slow recycling of various membrane proteins, such as AMPA receptor, rhodopsin, EGFR, TLR4, β1 integrin, *N*-cadherin, and E-cadherin (Kelly et al., 2012). In the following paragraphs, we will describe the slow recycling pathway, which comprises two continuous processes: EE-to-RE vesicle trafficking and RE-to-PM vesicle targeting.

In EE-to-RE trafficking, Rab10, Rab11, Rab22, and Rab25 are reported to be associated with this process as depletion or expression of dominant-negative forms of these proteins showed a decreased number of REs or inhibited RE biogenesis in diverse cell types (Wang et al., 2000; Weigert et al., 2004; Chen et al., 2006; Barral et al., 2008). For example, it seems that Rab11 and its effectors, such as Rab11 family interacting proteins (Rab11-FIPs) and microtubule motor proteins, are associated with EE-to-RE trafficking (Welz et al., 2014). Specifically, the Rab11 family interacting protein3 (Rab11-FIP3) complex was shown to directly interact with dynein light intermediate chain 1 (DLIC-1) and disruption of FIP3 binding with DLIC-1 inhibited EE-to-RE trafficking of TfR in epidermal carcinoma human cells (Horgan et al., 2010).

Vesicle targeting from REs to PM is achieved by the cooperation of Rab8 and Rab11. According to a previous study in hippocampal CA1 neurons, Rab11 translocates AMPA receptor-containing vesicles from the dendritic shaft to the dendritic spine. Then, Rab8 directly drives the insertion of AMPA receptor-containing vesicles into the synaptic membrane (Brown et al., 2007). This process is known to involve the actin cytoskeleton, which facilitates the movement of these vesicles. Myosin-Vb (MyoVb), an actin motor protein that can form a complex with Rab11 and FIP-2, directly mediates RE-to-PM vesicle transport (Wang et al., 2008). For tethering of vesicles coming from REs to PM, the interaction between Rab11 and the exocyst complex is required. One of the Rab11 effectors, Sec15, plays an important role in this process (Zhang et al., 2004). Once Sec15 binds to Rab11, they initiate sequential recruitment of exocyst complex subunits including cytoplasmic Exo84, Sec5, Sec6, Sec8, and Sec10, and PM-attached Exo70 and Sec3 (Zhang et al., 2004; Heider and Munson, 2012). They directly link the vesicle membrane and PM to promote targeted fusion of Rab11 vesicles with the PM.

#### Evidence for the Regulatory Roles of Rabs in Dendrite Morphogenesis

One of the best examples of experimental evidence for the involvement of early endosomal Rabs in dendrite morphogenesis comes from a study by Satoh et al. (2008), who showed in *Drosophila* class IV da (C4 da) neurons that mutation of a dynein subunit gene, *dlic*, led to proximally “bushy” dendrites and that *dlic* and *Rab5* double mutation resulted in greatly simplified dendritic morphology. Interestingly, this double mutant phenotype was similar to those seen in neurons with *Rab5* mutation only. These data indicate that Rab5, in a co-operation with *dlic*, plays a regulatory role in dendrite morphogenesis. Another study on the genetic interaction between *Protein Kinase A* (*PKA*) and *Rab5* in C4 da neurons showed that *PKA* could also contribute to the dendritic arbor development by altering Rab5-endosomal transport in dendrites (Copf, 2014). More recently, it was reported that BDNF-induced dendritic branching accompanied increased number and mobility of TrkB-positive Rab5-endosomes in cultured rat hippocampal neurons (Moya-Alvarado et al., 2018). Accordingly, expression of the dominant-negative form of Rab5 reduced dendritic arborization which was partially rescued by BDNF treatment.

Many studies also described the association between Rab35 and Rab4 with dendrites. Rab35 was shown to recruit a series of effectors, such as MICAL-L1, ACAP2, and EHD1, to inactivate ARF6 (Kobayashi and Fukuda, 2013) and promote vesicle targeting from REs to neurite tips, thereby inducing neurite outgrowth in PC12 cells (Kobayashi et al., 2014). Rab4-positive endosomes have been associated with the dendritic formation in *Drosophila* C4 da neurons (Zheng et al., 2008). They showed that *dlic* mutants induced proximal shift in both Rab4-positive endosomes and dendritic branch distribution. However, *dlic* mutants also altered localization of GOPs, suggesting that the proximal shift in the branch distribution may be, at least in part, due to the mislocalization of both Rab4-positive endosomes and GOPs. Also, Rab4 is reported to collect its neuron-specific effector GRASP-1 to co-ordinate RE maturation, which is necessary for surface expression of AMPA receptor in dendrites of cultured rat hippocampal neurons (Hoogenraad et al., 2010). A more recent study showed that Rab4 forms a complex with GluN2B and VPS35 to regulate the surface expression and recycling of GluN2B-NMDA receptor in dendrites of cultured mouse hippocampal neurons (Gromova et al., 2018). In this process, active Rab4 collects Neurobeachin (NBEA), a Brain-enriched multi-domain protein, to link the complex with motor protein KIF21B, which enables vesicle trafficking. Deficiency of either NBEA or KIF21B results in decreased actin enrichment in dendritic spines and consequent reduction of dendritic spine number.

REs have been studied extensively in neuronal dendrites; Rab11 is the most prominent RE-associated molecule. The role of Rab11 in dendrites was initially highlighted by a collection of research from the same group (Park et al., 2004, 2006), who showed that LTP-inducing stimuli promoted the mobilization of Rab11-REs towards dendritic spines and vesicle fusion with PM, which resulted in rapid spine growth in hippocampal neurons. Moreover, expression of the dominant-negative form of Rab11 decreased total spine numbers, whereas overexpression of wild-type Rab11 increased them (Park et al., 2006). More recent studies have shown the involvement of Rab11-REs in dendritic pruning in *Drosophila* C4 da neurons (Kramer et al., 2019; Lin et al., 2020). These studies suggest that appropriate localization of Rab11-REs in dendrites is crucial for dendritic PM turnover and morphogenesis.

The function of Rab10 has also been associated with dendrite morphogenesis in *C. elegans* (Taylor et al., 2015; Zou et al., 2015). Taylor et al. (2015) reported that *Rab10* mutants showed a reduction in posterior dendritic branches, but an increase in distal anterior branches in PVD neurons, indicating that Rab10 is a critical regulator of dendrite morphogenesis and patterning in *C. elegans* PVD sensory neurons.

Although these studies provide substantial evidence supporting the involvement of Rabs in dendrite morphogenesis, the mechanism by and the effectors with which they regulate dendritic PM turnover remain unclear. Further studies clarifying the exact regulatory roles of Rabs in dendrite morphogenesis would enrich our understanding of the physiological roles of the entire endosomal pathway in neurons.

#### Evidence for the Involvement of Local Rab-Mediated Endocytic Pathway in Dendritic Pathology in NDs

Several previous studies provide experimental evidence to support a link between endosomal defects and neuronal pathology in NDs, which has been well-reviewed in recent articles (Kiral et al., 2018; Guadagno and Progida, 2019). For example, in postmortem brains of AD patients, enlargement of Rab5-positive EEs and upregulation of Rab4 were observed in pyramidal neurons of the prefrontal cortex at the early-stages (Cataldo et al., 2000), and upregulation of Rab4, Rab5, Rab7, and Rab27 was observed in the cholinergic basal forebrain neurons (Ginsberg et al., 2011). In line with this, in animal models of HD, impaired conversion from Rab11-GDP to Rab11-GTP, and delayed TfR recycling back to PM were observed in primary cortical neurons (Li et al., 2009). Besides these general links between Rab-mediated endocytic pathway and dendritic pathology in NDs, more direct evidential links have been reported in studies using animal models of NDs. Umeda et al. (2015) reported that intracellular Aβ oligomers impaired endocytic vesicle trafficking of TfR in dendrites, which resulted in dendritic spine alteration in mouse primary neurons. Richards et al. (2011) showed that cultured hippocampal neurons expressing mutant huntingtin (htt) displayed a loss of dendritic spines when they were in proximity to htt aggregates and that this loss was due to functional defects in Rab11-mediated local endosomal recycling caused by the aggregates. Also, a previous study showed that the loss-of-function of TDP-43 in primary hippocampal neurons reduced the number and motility of Rab11-positive REs regulating NRG1-ErbB4-mediated trophic signaling in dendrites, thereby inducing dendritic defects (Schwenk et al., 2016). Another study showed that overexpression of mutant *CHMP2B*, which is associated with Frontotemporal dementia (FTD), in primary cortical neurons increased dendritic branches and decreased endolysosomal trafficking in dendrites (Clayton et al., 2018). Although the link between endosomal defects and dendritic pathology in a subset of NDs has been characterized as shown above, further studies on the details of underlying pathogenic mechanisms warrant further scrutiny.

### COPI and COPII as Potential Local Regulators of the Secretory Pathway

No matter which dendritic secretory pathway is being considered, the early secretory pathway (from ER-to-Golgi or ER-to-ERGIC) seems to be involved. In this section, we will briefly outline the generalized characteristics of the early secretory pathway by describing some of its key regulators and make extensions to the dendritic secretory pathway and NDs where appropriate.

#### Regulation of COPII Vesicle Budding and Fusion in the Early Secretory Pathway

The secretory pathway comprises the transport of secretory and membranous materials from ER to Golgi and ultimately to PM. ER-to-ERGIC and ERGIC-to-Golgi in mammals and ER-to-Golgi transport in other less-developed species such as *Drosophila* and yeast are mediated by coat protein complex II (COPII) vesicles (Brandizzi and Barlowe, 2013). The COPII pathway is initiated from the ER exit site (ERES), a site on the ER that lacks ribosomes, which is defined by the presence of Sec16 (Hughes et al., 2009) anchored there by leucine-rich repeat kinase 2 (LRRK2; Cho et al., 2014). Sec16 recruits Sec12 (Montegna et al., 2012), a GEF for Sar1 (Barlowe and Schekman, 1993). Sar1, in turn, recruits the inner COPII components (Sec23–Sec24 complex; Matsuoka et al., 1998). Next, the outer COPII components (Sec13–31 complex) are recruited to and bind at the interface of the Sar1-Sec23 complex (Bi et al., 2007; Fromme et al., 2007). These inner and outer COPII components ultimately induce GTP hydrolysis of Sar1, which leads to the scission of COPII vesicles from the ER (Bielli et al., 2005; Fromme et al., 2007). Immediately after scission, the vesicles uncoat prior to fusion with the ERGIC or *cis-*Golgi (Suda et al., 2017). This fusion process is mediated by Rab1 GTPase on COPII vesicles and GM130 on the membranes of ERGIC or *cis-*Golgi (Sztul and Lupashin, 2009).

#### Regulation of COPI Vesicle Budding and Fusion in the Early Secretory Pathway

The transport process between the ER and Golgi is not unidirectional. The best characterized retrograde transport process from Golgi to ER is the COPI pathway (Spang, 2013; Arakel and Schwappach, 2018). COPI comprises γ-COP–δ-COP–ζ-COP–β-COP tetrameric complex and α-COP–β′-COP–ε-COP trimeric complex that forms inner and outer layers of the COPI coat, respectively (Eugster et al., 2000). These complexes are recruited to the Golgi membrane upon activation of the ADP-ribosylation factor (ARF). Once recruited to the Golgi membrane, the subunits α-COP, β′-COP, γ-COP, and δ-COP recognize specific motifs on cargoes and promote their incorporation into COPI vesicles (Cosson and Letourneur, 1994; Brandizzi and Barlowe, 2013). The scission of COPI vesicles is mediated by dimerization of ARF1 (Beck et al., 2008, 2011), and its GTP hydrolysis promotes the uncoating of COPI vesicles (Tanigawa et al., 1993) before fusing with the ER membrane *via* Dsl1 tethering complex in yeast (Andag and Schmitt, 2003; Ren et al., 2009) and likely the NAG-RINT1-ZW10 (NRZ) complex in mammals (Hirose et al., 2004; Civril et al., 2010). However, whether these processes are conserved in the dendritic secretory systems in neurons remains unclear.

#### Evidence for the Involvement of Dendritic Secretory Pathway in Dendritic Pathology

Although the origins of dendritic secretory units are mostly unknown, we suspect that they are not entirely discrete from the canonical secretory units in the soma. Indeed, a study reported that GOPs may originate from somatic Golgi in rat hippocampal neurons (Quassollo et al., 2015). Interestingly, functional and structural alterations of somatic Golgi, termed Golgi pathology (Gosavi et al., 2002; Liazoghli et al., 2005; van Dis et al., 2014), as well as impaired exocytosis mediated by the secretory pathway (Larsen et al., 2006; Spencer et al., 2016), has been frequently observed in neurons of animal models for NDs. Provided that the dendritic secretory system has some reliance on the canonical secretory system, these evidences suggest a possibility of widespread involvement of the dendritic secretory pathway in dendritic pathology.

A recent study on polyQ toxicity in *Drosophila* has also provided a link between the dendritic secretory pathway and dendritic pathology. Chung et al. (2017) showed that nucleus-accumulated polyQ proteins led to the reduction of the *CrebA* mRNA level. Because CrebA is the master regulator of the secretory pathway (Abrams and Andrew, 2005; Fox et al., 2010), polyQ toxicity led to the perturbation of the COPII pathway, thereby decreasing GOP formation, and ultimately resulting in reduced dendritic branches (Chung et al., 2017). Indeed, knockdown of *Sec31* (Chung et al., 2017) or homozygotic mutation in *Sar1* in *Drosophila* da neurons (Ye et al., 2007) reduced the number or integrity of GOPs, respectively. The disruption also led to a significantly decreased dendritic PM supply, although to what extent GOPs, rather than somatic Golgi, contribute to such decrease is difficult to tell. Interestingly, when GOPs were selectively ablated by laser, dendritic branch dynamics were reduced (Ye et al., 2007). However, the extent to which the laser-ablated GOPs were not measured nor did the authors examine other potential damage that may have been induced by the laser.

Glutamatergic excitotoxicity involving the NMDA receptor is often observed in animal models of NDs (Lewerenz and Maher, 2015). Interestingly, NMDA receptor trafficking in dendrites is mediated by dendritic ERES and GOPs (Aridor et al., 2004; Jeyifous et al., 2009). This evidences suggest that excitotoxicity involving NMDA receptors may be dependent on the dendritic secretory pathway. Upon knock-out of *Lrrk2*, Sec16A detached from the dendritic ERES, which led to the impairment of ER-to-Golgi transport and NMDA receptor trafficking in mouse primary hippocampal neurons (Cho et al., 2014). Also, overexpression of PD-linked LRRK2 mutants has been shown to induce NMDA receptor-mediated excitotoxicity, leading to dendritic degeneration in rat cortical neurons (Plowey et al., 2014). These evidences support a model that suggests that the dendritic secretory pathway is regulated by LRRK2 whose dysfunction in PD is associated with NMDA receptor-mediated excitotoxicity and dendritic degeneration. Interestingly, Lin et al. (2015) found that Lrrk, a *Drosophila* ortholog of LRRK2, co-localized with somatic Golgi and GOPs in *Drosophila* da neurons, and that overexpression of a PD-linked mutant form of *LRRK2*, *LRRK2 G2019S*, suppressed anterograde movements of GOPs marked by ManII-eGFP. This GOP transport defect may underlie the dendrite degeneration observed in *LRRK2 G2019S*-expressing *Drosophila* da neurons (Lin et al., 2010). Whether or not other dendritic secretory units are also linked to NDs awaits further investigation.

## Conclusions and Perspectives

Neuronal dendrites seem to be highly vulnerable to neurotoxic insults, including those that arise in NDs (Luebke et al., 2010; Kulkarni and Firestein, 2012; Hasel et al., 2015; Kweon et al., 2017). This vulnerability may be partly due to differences between dendrites and soma in their response to stress, such as exposure to ROS or NMDA (Hasel et al., 2015). Here, we propose that dendritic endocytic and secretory pathways may be more susceptible than the canonical pathways to neurotoxicity, which could contribute to the vulnerability of dendrites in NDs.

Although the dendritic and the canonical pathways occur in distinct areas of the neuron, they share many of the regulatory molecules. Also, pieces of evidence show that at least parts of the dendritic secretory system, such as GOPs, may be derived from the canonical somatic secretory system (Quassollo et al., 2015), suggesting that the dendritic secretory system is under the purview of the canonical system in the soma. Thus, it is possible that when endocytic and secretory functions are under assault in neurons, the canonical system may need to limit its purview in dendrites to support its somatic functions. We posit several reasons in support of this possibility: (1) knockdown of *Sec31* and nuclear polyQ expression lead to the loss of GOPs, but not somatic Golgi (Chung et al., 2017); (2) loss-of-function mutations of genes related to ER-to-Golgi trafficking, such as *Sec31*, *Rab1*, and *Sar1*, all lead to impaired arborization of dendrites, but normal morphology of axons in *Drosophila* da neurons (Ye et al., 2007); (3) a partial loss-of-function in Golgi SNARE protein Membrin causes neuron-specific dysfunctions and significantly impairs dendritic growth in a *Drosophila* model for progressive myoclonus epilepsy (Praschberger et al., 2017); (4) neurons often undergo dendritic degeneration before cell death in NDs (Klapstein et al., 2001; Jaworski et al., 2011; Fogarty et al., 2016); (5) shrinking dendritic area has been identified as an adaptive response to SCA1 toxicity (Dell’Orco et al., 2015); (6) dendrites in *Drosophila* motoneurons (Ryglewski et al., 2014) and da neurons (Shorey et al., 2020) have been shown to be dispensable for neuronal survival; and (7) endocytic and secretory dysfunctions are often observed in a number of NDs (Wang et al., 2020). These results may partly explain the fact that neuronal dendrites are more vulnerable to neurotoxicity than other neuronal domains (Luebke et al., 2010; Hasel et al., 2015; Kweon et al., 2017). Further investigations in the dendritic endocytic and secretory pathways will be needed to test the validity of our hypothesis in addressing the issue of dendritic vulnerability in NDs.

In this review article, we presented our perspective view that impaired PM turnover involving dysregulation of the dendritic endocytic and secretory pathways may contribute to dendritic pathology in NDs. Although there is a growing body of evidence for the potential link between impaired PM turnover and dendritic pathology in NDs, our understanding of the exact pathogenic mechanisms remains largely elusive. We propose that dendritic pathology in NDs may involve dysregulation of the regulatory machinery, such as Rab GTPases and COPI/COPII, for the dendritic endocytic and secretory pathways described above. Dysregulation of the dendritic pathways appears to complement cytoskeleton impairment as underlying pathogenic mechanisms for dendritic pathology. Because dendritic defects are often early features of ND, future studies to elucidate the pathogenic mechanisms by which impaired PM turnover contributes to dendritic pathology in NDs will deepen our understanding of the early pathogenesis of NDs.

## Author Contributions

JP, CC, SP and SL conceptualized the theme of the review and wrote the manuscript together. All authors contributed to the article and approved the submitted version.

## Conflict of Interest

The authors declare that the research was conducted in the absence of any commercial or financial relationships that could be construed as a potential conflict of interest.
